# Whole body vibration therapy and diabetes type 2: a systematic review and meta-analysis

**DOI:** 10.3389/fendo.2024.1398375

**Published:** 2024-09-19

**Authors:** Juan Fabregat-Fernández, Vicente Rodríguez-Pérez, Rocío Llamas-Ramos, Ana Felicitas López-Rodríguez, María Cortés-Rodríguez, Inés Llamas-Ramos

**Affiliations:** ^1^ Department of Nursing, University of Extremadura, Plasencia, Spain; ^2^ Department of Nursing and Physiotherapy, Universidad de Salamanca, Salamanca, Spain; ^3^ Institute of Biomedical Research of Salamanca (IBSAL), Salamanca, Spain; ^4^ Department of Nursing and Physiotherapy, Faculty of Nursing and Physiotherapy, Universidad de León, León, Spain; ^5^ Department of Statistics, Universidad de Salamanca, Salamanca, Spain; ^6^ University Hospital of Salamanca, Salamanca, Spain

**Keywords:** whole body vibration, vibration platform, diabetes mellitus type 2, physical exercise, fasting blood glucose, glycosylated hemoglobin

## Abstract

**Background:**

Vibration platforms have demonstrated systemic effects generated by the use of mechanical vibrations, which are similar to those of any physical activity. The effect that whole body vibration (WBV) generates on the organism could be recommended in Diabetes Mellitus 2 (DM 2) patients.

**Objective:**

To systematically review and meta-analyze the available evidence on the effects of WBV on glycemic control in patients with DM 2

**Material and methods:**

Exhaustive bibliographic searches were carried out until October 2023 in different biomedical portals and databases: Public Medline (PubMed), Scientific Electronic Library Online (SciELO), VHL Regional Portal, Cochrane Central and Latin American and Caribbean Literature in Health Sciences (LILACS). Randomized clinical trials based on the effects of Whole Body Vibration on glycosylated hemoglobin levels, with control group and participants were non-insulin dependent were the inclusion criteria. Two reviewers extracted data independently. A third reviewer was available for discrepancies.

**Results:**

Six articles with 223 participants met the criteria and were included in the systematic review; only four of them met the criteria to be part of the meta-analysis. This meta-analysis reveals a positive and significant effect size (μ ê=0.5731), indicating a substantial difference between the groups studied. Although there is some variability between studies (heterogeneity of 30.05%), the overall direction of the effects is consistent. These findings conclusively suggest the presence of a significant influence of the variable evaluated, underscoring the robustness and consistency of the relationship observed in the literature reviewed.

**Conclusion:**

There are no conclusive results due to the lack of data for some variables, which prevents comparison; but WBV may be an effective therapy to improve glycemic control in DM 2 patients. More studies with more patients and longer follow-up are needed.

## 1 Introduction

Worldwide, 537 million people suffer from diabetes and this data will be increased up to 643 million by 2030 and 783 by 2045; in our country (Spain) the incidence of Diabetes Mellitus type 2 (DM 2) is estimated at 1 in 7 people, equivalent to 10.3% of the population (around 5.1 million adults). This situation generates a great impact on society, as well as concern among health professionals due to its high and ever-increasing prevalence, not to mention the high cost to the health system which could reach USD 966 billion dollars, which represents an increase of 316% over the last 15 years; raising awareness among these people is the key to combating this problem (1).

Several disciplines have made contributions to the current knowledge about DM 2; however, despite the passage of time, the fundamental pillars in the treatment of a diabetic person are based on diet and exercise, especially in DM 2. In addition, the physical disabilities and comorbidity present in most people with DM 2 are challenges to adherence to physical activity (2).

Due to the research during the last decades, the etiology, pathophysiological mechanisms, diagnosis and treatment of DM 2 are better understood. Traditionally, vibration exposure has been considered detrimental for causing harm in humans. However, several studies have shown beneficial effects of the application of whole-body vibration (WBV) with low frequencies, low amplitudes and short exposure times (3, 4) as a novel treatment for DM 2 patients (5).

The application of WBV has been associated with favorable changes in hormone levels, strength, power, muscle mass, muscle electrical activity, jumping ability, balance, psycho-physical health and activation at the cortical level, among others (6). Nevertheless, the effects of WBV on DM 2 are less well known.

Various systematic reviews have evaluated the impact of WBV on glycemic control in patients with DM 2. The results obtained are contradictory. While some studies, conducted in 2016 (7) and 2019 (8), suggest a slight improvement in glucose and HbA1c levels, another systematic review published in 2018 (9) questions the strength of this evidence, pointing out a low methodological quality in the included studies.

Nowadays most WBV studies published are focused on the level of neuromuscular dysfunction (10) and the muscular system. Muscles are known to react to vibration by contracting and stretching automatically. Vibration produces a muscle contraction reflex, called the tonic vibratory reflex (11–13). This reflex has been related to decreased pain threshold, increased blood circulation (14) increased hormone secretion, activation of the Golgi tendon organ and inhibition of antagonist muscles (4, 15–17).

The physiological modifications observed with WBV are analogous to those of any physical activity, having been described in addition to acute changes, chronic adaptations. Adaptation to physical exercise is what determines the positive changes that appear in our organism when performing any sport. The WBV effectiveness could be explained by the fact that, while with training work is performed on a certain number of tissues, with the use of mechanical vibrations the whole body is subjected to vibration, obtaining beneficial effects at a systemic level (16-118-19).

To summarize the current evidence, our objective was to perform a systematic review and meta-analysis of the effects of WBV intervention on blood glucose levels and glycosylated hemoglobin (HbA1c) levels in people with DM 2.

## 2 Materials and methods

### 2.1 Systematic literature research

An exhaustive literature search including randomized and controlled clinical trials obtained from the different biomedical portals and existing databases, from January 2019 to October 2023: Public Medline (PubMed), Scientific Electronic Library Online (SciELO), VHL Regional Portal, Cochrane Central Register of Controlled Trials (Cochrane CENTRAL) and Latin American and Caribbean Literature in Health Sciences (LILACS) has been conducted. Search terms included were *Type 2 Diabetes Mellitus*, *physical exercise*, *whole body vibration* and *glycosylated hemoglobin or HbA1c*.

The databases were searched using Boolean operators such as: “AND” and “OR”. Keywords have been combined with connectors in order to find valid articles for the aim of the present work. The “OR” connector has been used by joining the words that mean almost the same thing “exercise” and “Exercise on vibrating platforms”, and the “AND” connector has been used in order to give greater sensitivity and specificity to the search. No filters have been used and searches were made in “all fields” and “all index” (**Table 1**).

**Table 1:** Search strategy.

DATABASE	SEARCH STRATEGY
**PUBMED**	**(((((Type 2 Diabetes Mellitus) AND (physical exercise)) OR (exercise on vibrating platforms)) AND (whole body vibration)) AND (glycosylated hemoglobin)) OR (HbA1c)** ((((“diabetes mellitus, type 2”[MeSH Terms] OR “type 2 diabetes mellitus”[All Fields]) AND (“exercise”[MeSH Terms] OR “exercise”[All Fields] OR (“physical”[All Fields] AND “exercise”[All Fields]) OR “physical exercise”[All Fields])) OR ((“exercise”[MeSH Terms] OR “exercise”[All Fields] OR “exercises”[All Fields] OR “exercise therapy”[MeSH Terms] OR (“exercise”[All Fields] AND “therapy”[All Fields]) OR “exercise therapy”[All Fields] OR “exercising”[All Fields] OR “exercise s”[All Fields] OR “exercised”[All Fields] OR “exerciser”[All Fields] OR “exercisers”[All Fields]) AND (“vibrate”[All Fields] OR “vibrated”[All Fields] OR “vibrates”[All Fields] OR “vibrating”[All Fields] OR “vibration”[MeSH Terms] OR “vibration”[All Fields] OR “vibrations”[All Fields] OR “vibrational”[All Fields] OR “vibrator”[All Fields] OR “vibrators”[All Fields]) AND (“platform”[All Fields] OR “platform s”[All Fields] OR “platforms”[All Fields]))) AND ((“whole”[All Fields] OR “wholeness”[All Fields] OR “wholes”[All Fields]) AND (“human body”[MeSH Terms] OR (“human”[All Fields] AND “body”[All Fields]) OR “human body”[All Fields] OR “body”[All Fields]) AND (“vibrate”[All Fields] OR “vibrated”[All Fields] OR “vibrates”[All Fields] OR “vibrating”[All Fields] OR “vibration”[MeSH Terms] OR “vibration”[All Fields] OR “vibrations”[All Fields] OR “vibrational”[All Fields] OR “vibrator”[All Fields] OR “vibrators”[All Fields])) AND (“glycosylated haemoglobin”[All Fields] OR “glycated hemoglobin”[MeSH Terms] OR (“glycated”[All Fields] AND “hemoglobin”[All Fields]) OR “glycated hemoglobin”[All Fields] OR (“glycosylated”[All Fields] AND “hemoglobin”[All Fields]) OR “glycosylated hemoglobin”[All Fields])) OR (“glycated hemoglobin”[MeSH Terms] OR (“glycated”[All Fields] AND “hemoglobin”[All Fields]) OR “glycated hemoglobin”[All Fields] OR “hba1c”[All Fields] OR “hba1cs”[All Fields])
**SciELO**	(Type 2 Diabetes Mellitus) AND (physical exercise) OR (whole body vibration)
**VHL Regional Portal**	(Type 2 Diabetes Mellitus) AND (whole body vibration) (Type 2 Diabetes Mellitus) AND (physical exercise OR whole body vibration)
**Cochrane CENTRAL**	(Type 2 Diabetes Mellitus) AND (physical exercise OR exercise on vibrating platforms) AND (whole body vibration) AND (glycosylated hemoglobin OR HbA1c)
**LILACS**	(Type 2 Diabetes Mellitus) AND (whole body vibration) (Type 2 Diabetes Mellitus) AND (physical exercise OR whole body vibration) (whole body vibration) AND (glycosylated hemoglobin OR HbA1c)

PICO strategy has been followed as follows:

- POPULATIONS: diabetes type 2 patients

- INTERVENTIONS: whole body vibration therapy

- COMPARISONS: control groups

- OUTCOMES: fasting blood glucose, glycosylated hemoglobin

### 2.2 Selection criteria

Randomized controlled trials were considered eligible if they met inclusion criteria such as addressing the effects of WBV on glycosylated hemoglobin levels, the WBV intervention had to be at least 8 weeks, at least one control group did not perform WBV, and participants were non-insulin dependent. Exclusion criteria were studies in which individuals had a reported diabetic complication (neuropathy, retinopathy or diabetic peripheral nephropathy), animal studies and studies with insufficient description of WBV. Besides, a manual search was carried out based on the references of the selected articles as well as the references of other articles not included because they are a different type of study than the one selected in the present review.

### 2.3 Screening, selection and data extraction

Two independent reviewers in a first step carried out the bibliographic research and after that, examined the titles and abstracts of all studies identified through the search strategies. Studies that did not meet the inclusion criteria, based on titles or abstracts, were discarded. The two reviewers analyzed the full text of the remaining studies in a second review independently. If there is any disagreement a third reviewer was available to solve it.

Reviewers independently extracted data from the studies that met the inclusion criteria using a standardized data extraction form. Data extracted were: authors; year of publication; type of platform used; number of individuals forming the study sample; WBV parameters and intervention outcomes; mean and standard deviation of results.

### 2.4 Assessment of methodological quality and risk of bias

The physiotherapy evidence database (PEDro) scale was used to evaluate the methodological quality and the risk of bias of the randomized clinical trials included. This is a useful tool for assessing trial quality and the total score of this scale is 10 points. Punctuation more than 6 points is considered as a high-quality clinical trial. Sample selection, randomization, blinding (both participants and therapists), initial homogeneity and statistical analysis (intention to treat and comparisons) are included in this scale (20).

The quality of the included studies was scored by 2 investigators using the PEDro scale. The investigators rated the studies independently scoring from 0 to 10. If there is any disagreement a third reviewer was available to solve it.

Besides, the Van Heuvelen et al. (21) guidelines has been followed to report the quality of WBV studies. These guidelines encourage the authors to provide a detailed description of the WBV protocol applied (type of platform, frequency and amplitude of vibration, acceleration), the exposure parameters such as the duration and number of sessions as well as the total duration of the training program and the mode of application. In addition, the characteristics of the participants (demographic data, inclusion and exclusion criteria) should be presented. randomization and the existence of control groups should also be controlled, together with a presentation of the results and analysis of the data with statistical methods. finally, the existence of adverse effects and the existence or not of adherence, the interpretation of the results and the recommendations for clinical practice should be presented.

Besides, to test the quality of the meta-analysis the analysis implemented Random Effect Model using Jamovi software (22).

The *Random-Effect Model*: The analysis was carried out using the standardized mean difference as the outcome measure. A random-effects model was fitted to the data. The amount of heterogeneity (i.e., tau²), was estimated using the restricted maximum-likelihood estimator (23). In addition to the estimate of tau², the Q-test for heterogeneity (24) and the I² statistic are reported. In case any amount of heterogeneity is detected (i.e., tau² > 0, regardless of the results of the Q-test), a prediction interval for the true outcomes is also provided. Studentized residuals and Cook’s distances are used to examine whether studies may be outliers and/or influential in the context of the model. Studies with a studentized residual larger than the 100 x (1 - 0.05/(2 X k))th percentile of a standard normal distribution are considered potential outliers (i.e., using a Bonferroni correction with two-sided alpha = 0.05 for k studies included in the meta-analysis). Studies with a Cook’s distance larger than the median plus six times the interquartile range of the Cook’s distances are considered to be influential. The rank correlation test and the regression test, using the standard error of the observed outcomes as predictor, are used to check for funnel plot asymmetry.

## 3 Results

### 3.1 Study selection

**Figure 1:**
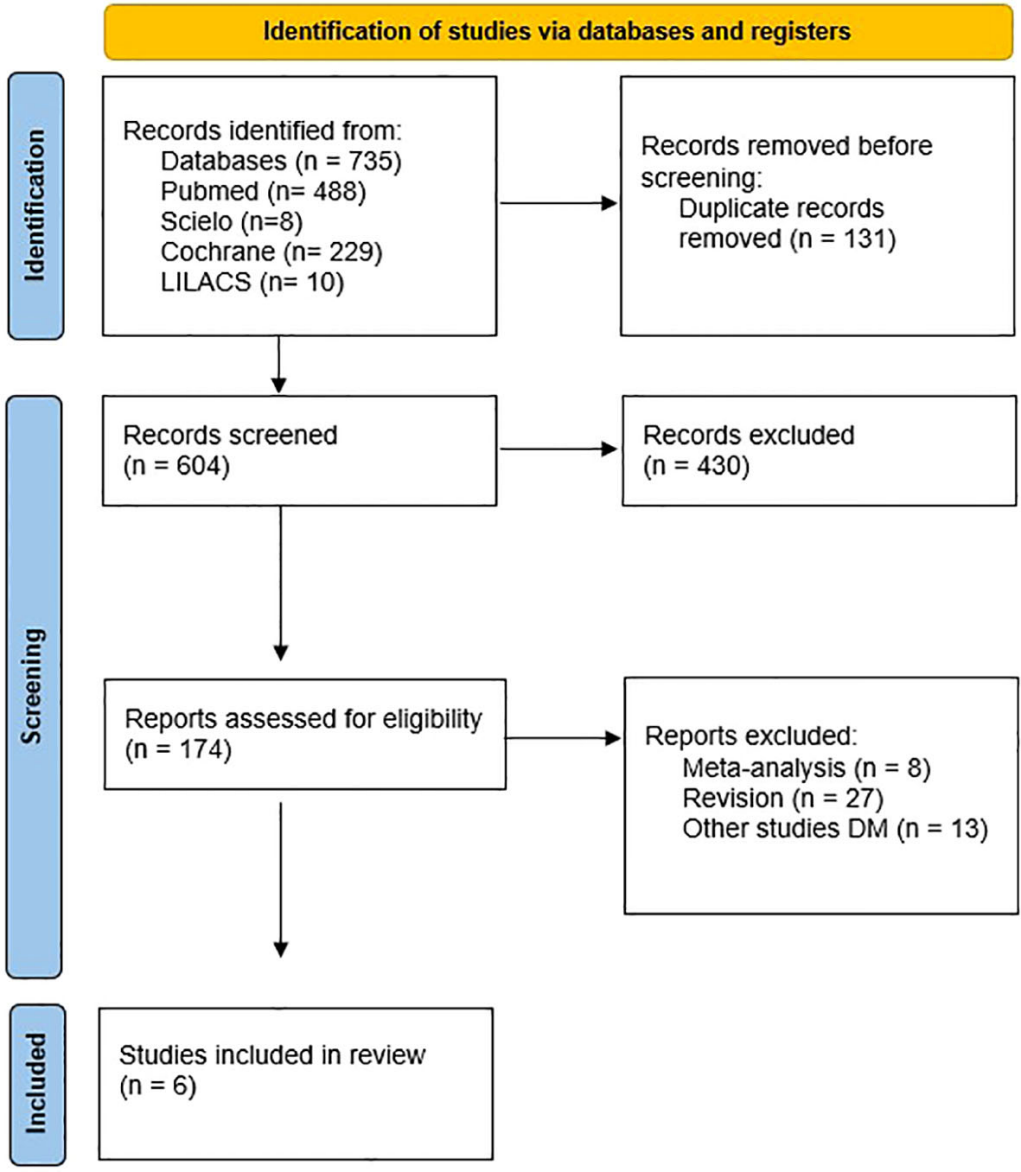
Study selection.

After the initial search, 735 potential studies were found to evaluate its possible inclusion in the systematic review. 131 manuscripts were removed as duplicates. The first screening by title and abstract reduced the sample to 174. A carefully full- text read of these files was made by reviewers and finally 6 records were included in the systematic review and 4 in the meta-analysis due the lack of some values in the two studies removed, which avoid comparisons. **Figure 1** shows the Prisma flowchart of the study selection.

### 3.2 Study characteristics

The sample analyzed in the articles reviewed ranged from 24 to 50 subjects, the mean of the sample being 37.1 patients. The selected articles show that 33.33% of the reviewed studies show the existence of a statistically significant decrease in glycosylated haemoglobin (HbA1c) after exposure to vibratory exercise (25–27). While for the remaining 66.66% (28, 29) there is no statistically significant decrease in HbA1c. Fasting blood glucose (FBG), however, decreased in 66.66% of the studies analyzed (25–27, 29), after the use of WBV, while for 33.33% (25, 30) there was no significant decrease.

There are basically 2 types of vibration platforms: vertical platforms or oscillating platforms. Vertical platforms vibrate in a predominantly vertical direction, moving vertically under both feet at the same time. Oscillating platforms vibrate around a horizontal axis, resulting in a simultaneous and symmetrical movement of both sides of the body during exposure (31, 32). The most commonly used platform in several studies (25, 26, 28), was the oscillating platform, obtaining positive results with respect to HbA1c and white blood cells (WBC). However, the use of the vertical platform (27), also concluded with positive effects on HbA1c and WBC results.

The duration of the intervention in the studies analyzed was between 8 (29) and 12 weeks (25–28, 30), with a mean of 11.33 weeks. Regarding the rest time between exercises applied in the interventions, it is located in an average of 34 seconds for those that reflect rest time between exercises (25–27). The other authors do not reflect rest time between exercises (28, 30). Most researchers propose an exercise frequency of 3 times per week, except Michels et al. (30) who carried out the intervention with 7 days of vibratory exercises. No study reports on possible adverse effects during the period of vibration work.

In **Table 2**, the main results of these articles were showed.

**Table 2:** Study characteristic of the selected articles.

AUTHOR	OBJECTIVE	PLATFORM	SAMPLE	INTERVENTION	RESULTS	CONCLUSIONS
**Manimmanakorn et al. (2017)** (25)	To identify beneficial changes for the health of people with DM 2 at WBV.	Fitvibe Excel (Vertical)	n = 36 WBVG (n=17) CG (usual physical activity) (n=19)	f 30Hz-2mm: 1 Wk. f 40Hz-4mm: 5-12 Wk. 3 x/Wk; 12 Wk 60sec VE/20 sec rest 12 min/session	↓ (p > 0,05) in FBG, HbA1c, insulin level and insulin sensitivity in both groups.	WBV may be an effective method to control some of the deleterious outcomes of DM 2 only in the most severe cases. No adverse effects.
**Alfonso-Rosa (2016)** (26)	To determine the applicability and effectiveness of WBV to improve functional capacity and quality of life in subjects with DM 2.	Physio Wave 700 (Oscillating)	n = 39 WBVG (n=19) CG (n=20)	f 12, 14 and 16Hz- 4mm 3 x/Wk; 12Wk. 30-45-60 sec VE (↑ progressive)/30 sec rest 8-16 min/session	↓ (p < 0,05) HbA1c and FBG WBVG CG ↓ HbA1C and FBG	↓ Glycaemia level in a VE session. No adverse effects.
**Del Pozo-Cruz et al. (2013)** (27)	To test the feasibility, safety and effectiveness of a 12-wk WBV intervention on glycemic control, lipid-related cardiovascular risk factors and functional capacity among DM 2 patients in a primary care context.	Physio Wave 700 (Oscillating)	n = 50 WBVG (n= 25) CG (n=25)	f 12, 14 and 16 Hz - 4mm 3 x/Wk; 12Wk. 30-45-60 sec VE (↑ progressive)/30 sec rest 10 min/session	↓ (p < 0,05) HbA1c and FBG WBVG and CG	VE is feasible, safe and effective in improving glycemic profile. No adverse effects.
**Baum et al. (2007)** (28)	**I**nfluence of AE and vibration on glucose metabolism parameters in people with DM 2.	Vibrogym Professional ^©^ (Oscillating)	n = 40 WBVG (n=14) Flexibility training group (8 static exercises)(n=13) Strength training group (8 stations in weight machines) (n=13)	f 30Hz-2mm: de la 1-9 Wk. f 35Hz-2mm: 10-12 Wk 3 x/Wk; 12Wk 30 sec EV/no rest 20 min/session	↓ (p < 0,05) in FBG for all groups. HbA1c ↓ (p > 0,05) in WBVG. ↑ HbA1c for other groups.	VE can be an effective and time-efficient tool for improving glycemic control in people with DM 2, although no significant data were obtained. No adverse effects.
**Behboudi et al. (2011)** (29)	Comparing how AE and WBV affect glycemic control in DM 2.	Star Sport-Taiwan (Oscillant)	n 30 AEG (n=10) WBVG (n=10) CG (n=10)	f= 30Hz-2 mm 3 x/Wk; 8 Wk 60 sec EV/60 sec rest 16 min/session (1-3 week) 20 min/session (4-6 week) 24min/session (7-8 week)	No effect for FBG for HbA1c. ↓ higher for AE and WBV than CG	Insignificant results due the sample size. WBV could be an option for DM 2 patients with obesity or those who cannot implement active physical activity No adverse effects.
**Michels et al. (2021)** (30)	To evaluate the effect of whole-body vibration at 28 Hz on glycemic control and other metabolic parameters in adults with DM 2.	SmartWalk (Vertical)	n = 22 WBVG (n= 11) CG (n=12)	f 28 Hz mm (no data) 7 x/Wk; 12Wk. 20-30 min/session No rest	HbA1c ↓ (p < 0,05) in WBVG FBG increase in both groups but there are no significant changes	Daily use of the vibration platform for 12 weeks improved HbA1c in adults with DM 2.

n, Sample size; WBV, whole body vibration; WBVG, whole body vibration group; CG, Control Group; DM 2, Diabetes mellitus type 2; FBG, fasting blood glucose; AEG, aerobic exercise group; Wk, week; Hz, Hertz; VE, vibration exercise; HbA1c, Glycosylated Hemoglobin; f, Frequency; AE, aerobic exercise; mm, millimeters; min, minutes.

Manimmanakorn (25) implemented a research with 36 patients with DM 2 divided into two groups. The WBV group performed for 3 times per week, 2 sets of 6 one-minute vibrating squats for 12 weeks and the control group performed their usual physical activity. These six positions were “(a) a deep squat position (knee angle 90o), (b) high squat position (knee angle 125o), (c) high squat position (with raised heels), (d) slight knee flexion 1 (holding hand straps with shoulder flexion), (e) slight knee flexion 2 (holding hand straps with shoulder abduction) and (f) slight knee flexion 3 (holding hand straps with elbow flexion)”. It was established a progression to 40 Hz and 4mm. They found no significant differences in HbA1c or FBG, concluding that WBV did not improve glycemic indices.

Alfonso-Rosa (26) published an article with 19 subjects with DM 2 exposed to a training based on 8 static and dynamic exercises with an elastic band (warm-up exercise: squat, up and down once with each foot, lunge, heel lift, squat, squat with weight changes, squats held with elastic bands, squat with elastic bands and side with elastic bands) on a vibrating platform at different frequencies (12, 14 and 16 Hz) while the control group continued with their usual activity. After 12 weeks it can be concluded that WBV produces an acute decrease in plasma levels, and the application of WBV at low frequencies is shown to be an effective and safe technique for the co-treatment and management of DM 2.

In this line, Del Pozo Cruz et al. (27) carried out a study with 50 subjects diagnosed with DM 2 were studied to test the feasibility, safety and efficacy of a whole body vibration intervention for 12 weeks in a primary health care setting performing 8 exercises in Physio Wave 700 oscillating platform (lunge, step up and down, squat, calf raises, left and right pivot, shoulder abduction with elastic bands, shoulder abduction with elastic bands while squatting, arm swinging with elastic bands) and the control group followed standard care. They establish a progression from 30 seconds for exercises and 30 seconds rest during the first month, while during the second and third months the exercise duration increased to 45-60 seconds and 30 seconds of rest and 2 more Hz each 4 weeks. They found a reduction in HbA1c and FGB compared to the control group concluding that the application of WBV in primary care is feasible, safe and effective in improving the glycemic profile.

Besides, Baum et al. (28) conducted another investigation with 40 non-insulin-dependent adult patients divided into 3 groups analyzed during 12 weeks of training with 3 training sessions per week. One group followed the WVB training with 30 Hz and 2mm from 1 to 9 week and 35 Hz the last weeks, the second, the strength group did the leg extension, seated leg flexion, leg press, seated calf raise, lat pulley, horizontal chest press, butterfly, and rowing exercises (1 set the first 6 weeks and 2 sets the last weeks) and the third group, flexibility group (control group) implemented 8 static exercises (the same progression was done increasing from 1 to 2 sets the last weeks). The main findings were that FBG remained unchanged after training and HbA1c tended to decrease below baseline in the vibration training group, while they increased in the other two intervention groups. Besides, the glucose tolerance improvement in WBV and strength groups. The authors concluded that these results suggest that vibration exercise may be an effective and low time-consuming tool to control glycemic control in patients with DM 2.

Behboudi et al. (29) selected a sample, 30 diabetic males, who followed a vibration exercise during 8–12-minute sessions of standing and semi-sitting positions at a frequency of 30 Hz and an amplitude of 2 mm, for 8 weeks three times a week. The aerobic exercise implemented 3 walking sessions a week and control group continued their routine activities. They detected no significant differences in HbA1c concentrations or fasting WBC between the conventional aerobic exercise groups and those subjected to vibration.

Finally, Michels et al. (30) investigated 22 adults with DM 2 who were taking oral antidiabetic agents were divided into 2 groups to submit one of them to 12-weeks intervention of WBV and control group received tips on how to change their lifestyle. After 12 weeks of intervention, they found a significant reduction in HbA1c in the WBV group, but no significant differences in FGB between groups.

### 3.3 Methodological quality and risk of bias

PEDro scale shows the quality of the randomized clinical trials. In this review, three articles showed high quality with 6, 7 and 9 points. Nevertheless, low methodological quality was shown in the other three articles with scores of 4, 5 and 5 points. In **Table 3**, the PEDro Scale assessment of the six selected articles is presented. Given the variability of the data presented in each manuscript and the lack of data for comparison with the other articles in the review, two manuscripts were removed from the meta-analysis.

**Table 3:** PEDro score of the randomized clinical trials selected.

AUTHOR	1	2	3	4	5	6	7	8	9	10	SCORE
**Manimmanakorn (2012)** (15)	Yes	No	Yes	No	Yes	Yes	Yes	Yes	Yes	Yes	9/10
**Alfonso-Rosa (2016)** (16)	Yes	No	Yes	No	No	No	No	Yes	Yes	Yes	5/10
**Del Pozo-Cruz et al. (2014)** (17)	Yes	No	Yes	No	No	No	No	Yes	Yes	Yes	5/10
**Baum et al. (2007)** (18)	Yes	No	Yes	No	No	No	Yes	Yes	Yes	Yes	7/10
**Behboudi et al. (2011)** (19)	Yes	No	Yes	No	No	No	Yes	No	Yes	No	4/10
**Michels et al. (2021)** (20)	Yes	No	Yes	No	No	No	Yes	Yes	Yes	Yes	6/10

#### 3.3.1 Quality of studies

Considering Van Heuvelen guidelines most of the articles have not adhered to these lines, so the results should be interpreted with caution (**Table 4**).

**Table 4:** Van Heuvelen et al. guidelines.

	ITEM	Manimmanakorn et al. (25)	Alfonso-Rosa et al. (26)	Del Pozo-Cruz et al. (27)	Baum et al. (28)	Behboudi et al. (29)	Michels et al. (30)
**Device**	Device specifications	Yes	Yes	Yes	Yes	Yes	Yes
	Platform constructions	No	No	No	No	No	No
**Vibration**	Type of vibration	Yes	Yes	Yes	Yes	No	No
	Vibration parameters	Yes	Yes	Yes	Yes	Yes	Yes
	Parameters verification	No	No	No	No	No	No
	Side-alternating vibrations: accelerometer location	No	No	No	No	No	No
	Frequency and magnitude: constant or modulated	Modulated	Modulated	Modulated	Constant	Modulated	Constant
**Administration**	Posture/body position	Yes	Yes	No	Yes	Yes	Yes
	Feet position	Partly	Yes	No	Yes	No	Yes
	Feet skidding prevention	No	No	No	No	No	No
	Head vibration transmission prevention	No	No	No	No	No	No
	Handrail	No	No	No	No	No	No
	Hands position	Partly	Partly	No	Partly	No	No
	Body parts subjected to vibration	Yes	Yes	Yes	Yes	Yes	Yes
	General exercise parameters	Yes	Yes	Yes	Yes	No	Yes
**Protocol**	Setting of sessions	Yes	Yes	No	No	No	No
	Trainer	No	No	No	Yes	No	No
	Previous instructions	Yes	Yes	Yes	No	No	No
	Preparatory exercises	No	No	Yes	No	Yes	No
	Subjects’ footwear	No	No	No	No	No	No
	Control intervention	Yes	Yes	Yes	Yes	Yes	Yes
	Time of outcome measurement	Yes	Yes	Yes	Yes	Yes	Yes
**Subjects**	General characteristics	Yes	Yes	Yes	Yes	Yes	Yes
	Previous experience	No	No	No	No	No	No
	Acute, short-term or long-term effects	Acute	Acute	Acute	Acute	Yes	Acute

#### 3.3.2 Random-effect model

**Figure 2:**
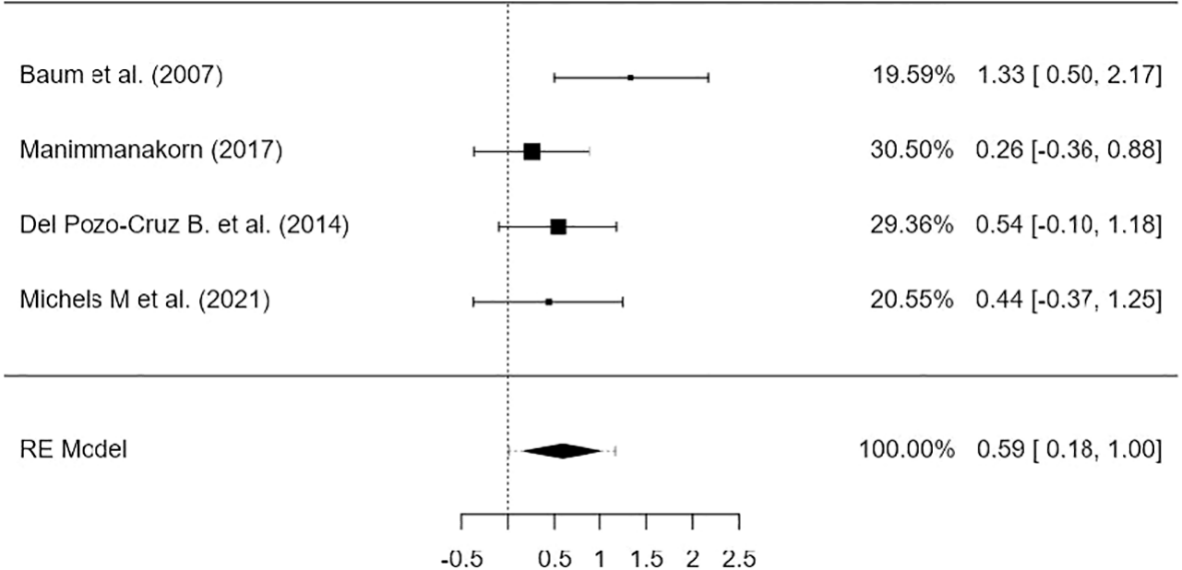
Forest plot.

A total of k=4 studies were included in the analysis. The observed standardized mean differences ranged from 0.2582 to 1.3338, with most estimates being positive (100%). The estimated average standardized mean difference based on the random-effects model was \hat{\mu} = 0.5895 (95% CI: 0.1782 to 1.0008). Therefore, the average outcome differed significantly from zero (z= 2.8088, p = 0.0050). According to the Q-test, there was no significant amount of heterogeneity in the true outcomes (Q (3) = 4.2890, p = 0.2319, tau² = 0.0436, I² = 24.6411%). A 95% prediction interval for the true outcomes is given by 0.0092 to 1.1698. Hence, even though there may be some heterogeneity, the true outcomes of the studies are generally in the same direction as the estimated average outcome. An examination of the studentized residuals revealed that none of the studies had a value larger than ± 2.4977 and hence there was no indication of outliers in the context of this model. According to the Cook’s distances, none of the studies could be considered to be overly influential. Neither the rank correlation nor the regression test indicated any funnel plot asymmetry (p = 0.3333 and p = 0.1512, respectively). The funnel plot asymmetry was not assessed because there are less than 10 articles (**Tables 5**, **6**, **Figure 2**).

**Table 5:** Random-effects model.

Random-Effects Model (k = 4)						
	Estimate	se	Z	p	CI Lower Bound	CI Upper Bound
**Intercept**	0.589	0.210	2.81	0.005	0.178	1.001

**Table 6:** Heterogeneity statistics.

Heterogeneity Statistics							
Tau	Tau^2^	I^2^	H^2^	R^2^	df	Q	p
**0.209**	0.0436 M (SE = 0.1438)	24.64%	1.327		3.000	4.289	0.232

## 4 Discussion

Whole body vibration is a simple, safe and effective non-pharmacological measure to reduce HbA1c and plasma glucose levels in patients with DM 2 whose physical condition prevents conventional aerobic exercise (33).

Regarding the pharmacological drugs for the treatment of DM 2, they do not inherently exhibit the ability to maintain sustained glycemic control over a prolonged period (34). In addition, adverse effects such as gastroenteritis, hypoglycemia and weight gain, among others, are undesirable manifestations associated with the use of orally administered pharmacological agents in patients with DM 2. These side effects lead to a decrease in adherence to pharmacological treatment by patients diagnosed with DM2 (35, 36). For that reason, moderate-intensity aerobic physical exercise is recognized as an effective non-pharmacological approach to reducing blood glucose levels for people whose physical condition allows it to be performed. However, WBV could be a physical exercise option to reduce blood glucose and HbA1c levels for those who cannot perform aerobic exercise (26–29).

Regular physical exercise increases glucose uptake in activated muscle, which induces increased insulin sensitivity in diabetics. Regular WBV training has been shown to generate muscle adaptation similar to resistance exercise training (37). Thus, regular WBV training may help diabetic patients to control glucose metabolism (25).

WBV does not require a specific physical condition, nor prior supervision and may help in physical exercise adherence for patients with DM 2. Moreover, it has demonstrated its efficacy in animal (38–40) and human (16, 41, 42) studies. Some of the participants who underwent the WBV intervention showed a statistically significant reduction in HbA1c levels compared to those who did not receive the intervention.

Some studies (26, 27, 30) point out, it seems that 12 weeks of WBV intervention is sufficient for a statistically significant improvement in both WBC and HbA1c in people with DM 2 compared to those who did not undergo any intervention. This data is similar to those found in another meta-analysis (7) in which the sample was subjected to aerobic training for 12 weeks, achieving a reduction in HbA1c. On the other hand, authors stated that there is no significant differences in HbA1c concentrations or fasting WBC between the conventional aerobic exercise groups and those subjected to vibration (29).

However, there is no clear consensus on what the most beneficial parameters for these patients should be. Studies with longer duration and parameters of frequency, intensity and duration should be investigated in the future in order to adapt this therapy to patients with type 2 diabetes.

In this period, Baum et al. (28) and Manimmanakorn (25), using a step-up frequency of 30-40Hz, did not show a significant reduction in HbA1c which was only revealed after *post-hoc* dichotomization. However, Alfonso-Rosa (26) and Del Pozo-Cruz et al. (27), using a lower frequency, also of progressive increase (12-16Hz), did show a significant reduction in both HbA1c and WBC. These data coincide with another study (31), where an increase in the effects was demonstrated in relation to the progressive increase in frequency. The gradual increase in frequency may be the reason for the difference in the results. Therefore, it seems that a low frequency with progressive increase and the number of weeks of intervention play a determining role in obtaining a beneficial effect. On the other hand, Michels et al. (30), using a constant frequency of 28 Hz, showed a reduction in HbA1c and WBC. Behboudi et al. (29) using a constant high frequency of 30 Hz for a shorter number of weeks showed no significant differences in HbA1c or fasting glucose, but concentration was higher in the control group.

During exposure, the vibration of the vertical platform moves vertically under both feet at the same time, producing a simultaneous symmetrical movement of both sides of the body, whereas the oscillating platform generates an asymmetrical perturbation of the legs. Vertical platforms work at higher frequencies (between 30 and 50 Hz) than oscillating platforms (between 5 and 30 Hz). In addition, vertical platforms have shown greater chronic effects on strength training and oscillating platforms greater acute effects (31). However, the limited scientific literature on the use of these platforms in diabetes makes it difficult to determine which is the best choice. Regarding the type of platform used, different studies (26, 28) have carried out an intervention using an oscillating type of platform, obtaining positive results with respect to HbA1c and WBC, as in the study by Lythgo et al. (43). However, in one of the studies (25), using the same type of platform, they did not obtain positive results for HbA1c, but did obtain positive results for WBC.

On the other hand, two of the studies (27, 30) used a vertical type of vibrating platform and found positive effects on HbA1c and WBC results. These data agree with what was announced by another study (32) where they determined that vibration transmission was higher during vertical vibration compared to oscillating vibration. This could indicate that the type of platform alone is not determinant to assess its effectiveness, more conclusive studies are needed to evaluate the different vibratory devices in people with DM 2.

HbA1C is one of the most determinant parameters in the diagnosis and long-term control of DM. According to the American Diabetes Association (ADA) (44), an HbA1c > 6.5% is considered a diagnostic criterion for DM, while values < 7.0% HbA1c determine good blood glucose control in the last four months (45, 46). Unfortunately, most of the studies using the HbA1chave performed short-term follow-ups, which prevents us from knowing the physiological effects in the long term,

Based on the information offered by the ADA (44), which considers that maintaining HbA1c levels below 7% acts as a protective factor, helping to manage glycemic imbalance and evaluate the risk of developing, Manimmanakorn (25) found a slight reduction in HbA1c, after 12 weeks of intervention in subjects with values above 8% using a *post hoc* dichotomization; in contrast, in a previous study, in which intervention exercises were applied for 8 weeks, no significant improvement in HbA1c was observed (47).

In relation to the control group, no significant changes in HbA1c levels were observed, according to the data provided by Manimmanakorn (25). On the other hand, Baum et al. compared the WBV group with a strength and flexibility group. After the vibration intervention, a reduction in HbA1c levels was found in both groups, although not reaching statistical significance (28). However, other authors, such as Alfonso-Rosa (16), Del Pozo-Cruz et al. (27) and Behboudi et al. (29) indicated that a conventional exercise program resulted in a decrease in HbA1c levels after the intervention period.

These data are in line with those provided by other studies (48), which show how a conventional training program reduces HbA1c levels.

The WBV guidelines reported by Van Heuvelen et al. (21) are essential to improve reproducibility and transparency in WBV studies, and their adherence allows for proper assessment of the efficacy and safety of WBV interventions. Follow them ensures that the necessary criteria for robust and replicable research are met. In this sense, all the authors of the selected articles (25–30) have reported the frequency of the intervention, as well as the amplitude, except for Michels et al. (30), the duration and frequency of the sessions and the total duration of the program, which was established at 12 weeks, except for Behboudi et al. (29) who applied 8 weeks. The application of the therapy, the results, the statistical analysis and the absence of adverse effects in all the selected articles were described. However, adherence has been variable, with 4 losses reported by Manimmanakorn et al. (25), 11 by Alonso-Rosa et al. (26) and del Pozo-Cruz et al. (27), and 2 by Michels et al. (30); only Baum et al. (28) and Behboudi et al. (29) did not report any losses. The recommendations for clinical practice and future lines of research recommend stratifying patients in the sampling according to the degree of severity of diabetes based on HbA1c or the duration of the disease (25) and analyzing cost effectiveness (27) as well as including more patients and longer follow-up (30) and optimizing frequency, amplitude, and duration of vibration exercises (28).

Finally, there is no consensus with the conclusions: WBV is applicable, safe and effective in reducing HbA1C and basal blood glucose level (26–28, 30), while for others (25) there was no change in FBS or HbA1c, nor for Behboudi et al. (29) who proposed that insignificant results of the present study can be attributed to the small number of samples and improper time and intensity of exercise. However, whole body vibration can be considered as a better way to exercise in a shorter time for majority of diabetic patients who suffer from obesity and unwillingness to join active physical activities. Because of these results, the effect size of these interventions is small and the clinical implications, although they appear to be promising, should be interpreted with caution.

The review has several limitations. the review was not registered; however, all steps were taken to ensure the reliability, transparency and thoroughness of the process and data. Only full-text registries have been considered; the number of experimental clinical trials using WBV in these pathologies is small and some of the studies do not provide all the data on the variables used like disease duration or some outcomes which prevent the effect comparisons. One of the selected articles has not been submitted a per-review process, therefore, the results obtained in this manuscript should be treated with caution and most of them did not follow the quality guidelines and recommendations exposed by Van Heuvelen (21). In addition, the sample size has been very diverse and small; there is not a standard protocol or parameters to achieve the best effectiveness of this therapy although most of them agree with a frequency of three times a week for 12 weeks, and the follow-up of the studies has been only focused on the short-term effects of WBV. In addition, it should be noted that the treatments received by the patients of the selected studies were not composed only of WBV treatment, so that the sum of the effects of the prescribed pharmacological treatment and lifestyle may have contributed to the positive effects obtained.

Nevertheless, the clinical implications are promising, being a safe therapy with no adverse effects that can be used in this population as a complement to other therapies or as an alternative for patients who are unable to perform physical activity. In future lines of research larger samples should be recruited as well as similar measurements, platforms and long term follow up to be able to better determine the clinical effects of the use of WBV.

## 5 Conclusion

The habitual use of vibration platforms with a frequency of 14-16 Hz for 12 weeks is reflected in the literature. These results are encouraging and suggest that WBV may be an effective therapy to improve glycemic control in patients with DM 2. The addition of Whole Body Vibration therapy as a complement for exercise programs could be effective in sedentary people and could be a useful tool for clinicians. However, further studies with more patients and longer follow-up are needed to confirm these findings.

## Figures and Tables

**Figure:**
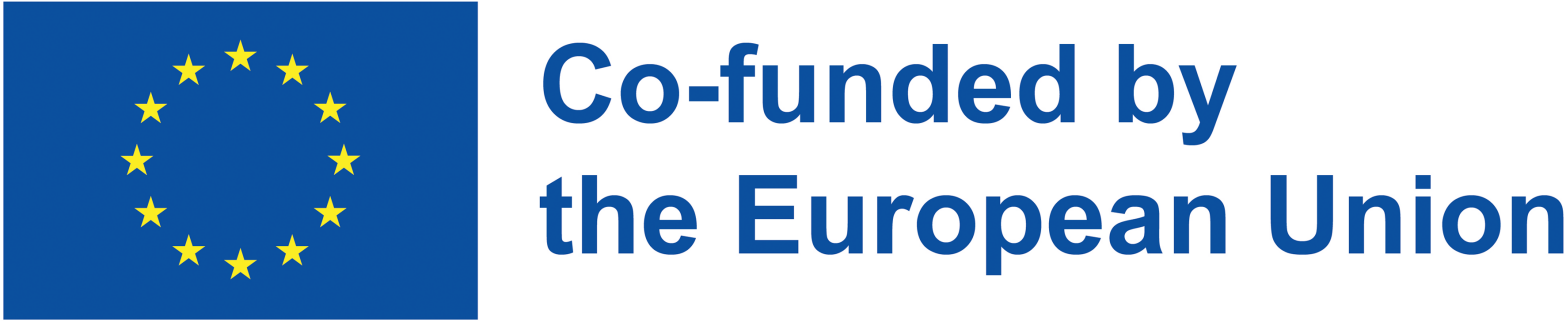


**Figure:**
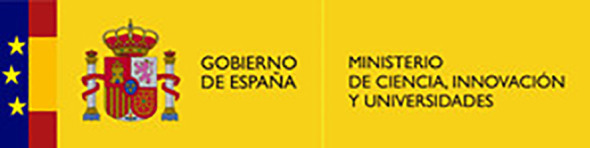


## Data Availability

The original contributions presented in the study are included in the article/supplementary material. Further inquiries can be directed to the corresponding author.
